# Fortifying of probiotic yogurt with free and microencapsulated extract of *Tragopogon Collinus* and its effect on the viability of *Lactobacillus casei* and *Lactobacillus plantarum*

**DOI:** 10.1002/fsn3.2250

**Published:** 2021-05-06

**Authors:** Mohammad Maleki, Peiman Ariaii, Mahdi Sharifi Soltani

**Affiliations:** ^1^ Department of Food Science and Technology Islamic Azad University, Ayatollah Amoli Branch Amol Iran; ^2^ Department of Veterinary Agriculture Faculty Islamic Azad University, Chalous Branch Chalous Iran

**Keywords:** functional foods, maltodextrin, probiotic yogurt, *Tragopogon Collinus*, ultrasound, whey protein concentrate

## Abstract

In this study, the effect of free and microencapsulation of *Tragopogon Collins* extract (TPE) on the properties of probiotic yogurt was investigated. For this purpose, first, TPE was extracted by ultrasound method. The amounts of phenolic and flavonoid compounds in TPE were 890.04 mg/g gallic acid and 512.76 mg/g extract (respectively), and it had high antioxidant and antimicrobial properties. Then, the extract was encapsulated by maltodextrin–whey protein concentrate. The results related to the particle size, zeta‐potential, and microencapsulation efficiency of the TPE microencapsulation were 93.87 nm, 18.99 MV, and 64.35% respectively. In order to investigate the effect of nano‐ and free TPE on the properties of yogurt during a 15‐day storage period of 5 treatments including control, nano‐ and free TPE at 750 and 1,000 ppm were provided and the physicochemical properties, probiotic bacteria viability, and sensory properties were investigated. The results showed that adding TPE to yogurt affects the physicochemical properties, probiotic bacterial viability, and sensory properties were investigated. The results showed that adding TPE to yogurt affects the physicochemical properties. TPE samples had lower pH, less syneresis, and more acidity, viscosity, and antioxidant properties compared to the control sample (*p* < .05). Furthermore, in these samples, the viability of probiotic bacteria during storage was higher than the control treatment and the sensory properties were acceptable. In most cases, better results were observed in nano‐TPE treatment. Therefore, by industrial production of probiotic yogurt containing nano‐TPE as a functional food, a new choice will be provided for consumers of dairy products that would have more desirable nutritional value and sensory properties.

## Graphical abstract

Functional foods contain one or more specific compounds that have a functional effect on improving the health and well‐being of the consumer. According to the results, it seems that the production of yogurt containing nano‐extract of TPE, as a functional food, provided a new choice for consumers of dairy products that, in addition to the desired taste, also have good nutritional properties.

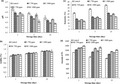

## 1 INTRODUCTION

Functional foods contain one or more specific compounds that have a functional effect on improving the health and well‐being of the consumer. These beneficial components may be naturally increased in the food or may be intentionally added during the production process to produce health effects such as regulating metabolic activities, fitness, improving the function of the digestive systems, heart, vessels, etc (Villaño et al., 2016). Probiotic products are one of the most common types of functional foods. In recent years, there has been an increasing effort to use probiotic microorganisms in the production of various foods in which they have been marketed, including probiotic yogurts, cheese, and fermented beverage (Keshavarzi et al., 2021). Probiotics not only do not cause any problem in the production process, but are also very effective in promoting the usefulness of the product and improving health of the consumers (Dols et al., 2011; Nyanzi et al., 2021; Prasanna Pradeep & Charalampopoulos, 2019). At the same time, probiotic products have received the legitimate attention of regulatory authorities with an interest in protecting consumers from misleading claims (Hill et al., 2014). The most common microbial cultures used in the production of probiotic products are the probiotic bacteria *Lactobacillus casei* (*L*. *casei*) and *Lactobacillus plantarum* (*L*. *plantarum*) (Yao et al., 2020). Currently, probiotic yogurt is the most accepted and widely used probiotic products in the world. Factors such as texture, taste, and acid production by starter bacteria in yogurt during fermentation time and even storage time can be improved by changing the process conditions or adding supplements to yogurt. These elements or supplements that determine the nutritional value or functional properties (or both) in yogurt include milk proteins, prebiotics, and herbs (Ghaleh Mosiyani et al., 2017).

Moreover, the increased resistance of pathogenic microbes in food to antibiotics is another concern. Therefore, there is a great tendency to utilize new types of natural antimicrobial and antioxidant compounds. Nowadays, the utilization of natural antioxidants such as herbal essential oils and extracts as a substitute for synthetic antioxidants is highly recommended to preserve food. Recently, research has focused on probiotic compounds in herbal extracts, but few studies have been done on the functional and probiotic properties of herbal extracts in yogurt (Oh et al., 2016; Valipour et al., 2017). An important plant in this case is *Tragopogon Collinus*.

*Tragopogon collinus* belongs to the Asteraceae family (Chicory). The compounds identified in these various plants are in the genus of flavonoids, terpenes, saponins, benzyl and hydroisocoumarin, phenolic compounds, and sterols, in which, many of them have been identified during chemotaxonomic studies of these plants. Some plants in the TPE genus, by their strong antioxidant effect, prevent inflammatory damage to tissues and in some concentrations prevent DNA damage (Farzaei et al., 2014).

The extraction of these bioactive compounds from the plant depends on several factors, which the most important of them are solvents and extraction methods. One of the new extraction methods is the use of ultrasound waves. This method is cheap, simple and effective, and additionally, increasing the extraction efficiency and speeding up the reaction is one of its most significant advantages. In this method, a lower temperature is required for extraction; thus, it causes less damage to heat‐sensitive compounds. Compared to other new extraction methods, this method is easier and cheaper and can be done with any type of solvent (Maleki et al., 2016).

The active ingredients of herbal extracts and essential oils are volatile, and some of them are difficult to dissolve in water and are also easily oxidized. One of the solutions to overcome these limitations is microencapsulation of the extract. Encapsulation is a process in which the fine particles and droplets of a substance are covered by different materials to obtain useful properties. One of the microencapsulation methods is to use a spray dryer. Some studies have shown that microencapsulation can increase the antimicrobial and antioxidant properties of compounds: moreover, maintain the stability of its properties for a longer period (Javadian et al, 2016; Bagheri et al., 2016). Coating materials include proteins, carbohydrates, lipids, gums, and cellulose. Maltodextrin is one of the most important and widely used carrier compounds due to its high solubility and low viscosity (Chranioti et al., 2015). Whey protein concentrate (WPC) is a suitable option for coating the carbohydrates microcapsules and has food and nonfood uses due to its special physicochemical properties, such as the possibility of using whey proteins as pH‐sensitive hydrogels in controlled release of microencapsulated bioactive compounds (Rajam et al., 2012).

According to what has been stated, evaluating the effect of free and microencapsulated of TPE (using maltodextrin–WPC) on physicochemical and sensory properties of probiotic yogurt and viability of *L. casei* and *L. plantarum* in this process is the aim of this study.

## 2 MATERIALS AND METHODS

### 2.1 Raw material

*Tragopogon Collinus* extract (TPE) from the genus of T. buphthalmoides is produced in summer areas of Chalous city, Mazandaran province, and it was approved by the Culture and Development Department of Tehran Medicinal Plants Institute. After identification, the excess parts were removed and dried immediately after washing. Then, it was dried in a vacuum vessel at 50°C for 45 min. It was completely pulverized by a shredder and kept at 25°C until the maintained time. All chemicals used were manufactured by Merck Company (Darmstadt, Germany) and were of analytical grade.

### 2.2 Extraction of the extract with ultrasound waves

10 g of TPE sample was extracted with 100‐ml ethanol: water (50:50%) at a temperature (45°C) and time (20 min) in an ultrasound bath at 20 KHz (Sonic and Material Vibra‐cell VC600; Sonic and Material Inc.). At the next level, the solutions were filtered through Whatman paper No. 1 and the solvents were evaporated under vacuum. The resulting extract was stored at −18°C until the experiment time (Maleki et al., 2016).

### 2.3 Measurement of phenolic and flavonoid compounds

Total phenolic compounds were determined based on the method described by Rashidaie Abandansarie et al. (2019), and it was determined based on Gallic acid by using a spectrophotometer (T80, UK).

Total flavonoids were determined by the aluminum chloride calorimetric method. 0.5 ml of the extract was added to 100 μl of 1 M potassium acetate, and after 5 min, 100 μl of 10% aluminum chloride was added to the solution. Then, 1.5 ml of 80% methanol and 2.8 ml of deionized water were added to the solution. After 30 min, the adsorption intensity of the solution was read at a wavelength of 415 nm. A standard curve diagram was drawn to calculate the total flavonoid concentration using routine, and the total flavonoids were presented in milligram in a gram of dry weight.

### 2.4 Measurement of antioxidant activity of the extract

#### 2.4.1 Investigation of free radical scavenging (DPPH)

For this purpose, 1 ml of different concentrations of the extract and nano‐extract was added separately (100, 500, and 1,000 ppm) with 1 ml of 0.1 mm DPPH solution and the resulting mixture was shaken well and placed in a dark room for 15 min. Then, the light absorption of the samples was read at a wavelength of 517 nm against the blank. All of these steps were performed on BHA as a standard antioxidant (Maleki et al., 2016).

(blank adsorption/blank adsorption−sample adsorption) × 100% = percentage of free radical scavenging DPPH.

#### 2.4.2 Measurement of ferric reducing antioxidant power (FRAP)

Measurement of strength to assay this property, 0.1 g of the extract is homogenized with 5 ml of distilled water in a cold porcelain mortar in an ice bath. The resulting homogenate was filtered using Whatman filter paper No. 1. Then, 1.5 ml of FRAP reagent (300 mM sodium acetate buffer with pH = 3.6, ferric‐triperidyl‐s‐triazine and ferric chloride) was added to 50 μl of the obtained extract. The resulting mixture was vortexed and incubated for 30 min at 30°C. The adsorption of the solutions was read at 593 nm compared to the blank (including 50 μl of distilled water with 1.5 ml of FRAP reagent). Ammonium ferrous sulfate was used as a blank for comparison (Tometri et al., 2020).

#### 2.4.3 Minimum inhibitory concentration and minimum lethal concentration of the extract

The liquid dilution method was used according to NCCLS recommendations. The studied (*Staphylococcus aureus*, PTCC 1112 and *Escherichia coli*, PTCC 1330) with an approximate concentration of 10^8^ cfu/ml were added to each of the test tube at a rate of 0.2 ml. In the next step, TPE solutions were prepared using Tween 80 (Merck, Germany) and distilled water in such a way that was produced by pouring 0.2 ml of each solution into the test tube containing liquid culture medium and the bacteria, which were under test. The test tubes were then incubated in the incubator at 37°C for the bacteria. After 24 hr, the lowest concentration was considered MIC that no turbidity was observed. In fact, the turbidity of the culture medium inside the erlens indicates the growth of bacteria, and the first test tube was considered the minimum inhibitory concentration (MIC) in which no turbidity was observed and was completely transparent. Under completely sterile conditions, after determining the MIC to determine the MBC of the erlens contents, which were still clear after 24 hr of incubation and no turbidity was observed, 0.1 ml in petri dishes containing a suitable culture medium for any bacteria was surface cultivated. After 24 hr of incubation at the appropriate temperature, the growth and nongrowth of bacteria were examined. The first nongrowth plate was considered MBC (Rashidaie Abandansarie et al., 2019).

### 2.5 Preparation of microencapsulated extract

Maltodextrin–WPC was selected as the carrier to prepare the microencapsulated TPE. Microencapsulation was prepared using the method of Noshad et al., (2015). The maltodextrin–WPC mixture was dissolved in chloroform/methanol solution (1:3 w:w). Then, the resulting solution was placed in a rotary evaporator to remove solvents to form a thin film on the wall. TPE was also dissolved in dichloromethane/methanol (1:2 w:w) solution, and the resulting mixture was mixed with maltodextrin–WPC in a ratio of 4:1 (maltodextrin–whey protein concentrate:extract) and the existing solvents were evaporated under nitrogen vapor. The produced film was dissolved in 2 ml of phosphate buffer (10 mmol/L, pH 7.4) and homogenized for 15 min at 35°C by a homogenizer at a pressure of 200 bar. The resulting suspension was placed in the dark at room temperature for 2 hr and then centrifuged at 4,500 *g* at 4°C. Finally, microencapsulated TPE was dried using a freeze dryer (Operon FDB‐550).

### 2.6 Microencapsulation efficiency

The microencapsulation efficiency of polyphenols was determined according to the method described by Robert et al., (2015). 200 mg of microencapsulated was added to 2 ml ethanol and stirred for one minute, and then it was subjected to ultrasound for 20 min in two stages with 100% intensity and 20 kHz frequency. After this step, centrifugation was performed at 3,000 *g* for 2 min. Alcohol can dissolve extracts outside the capsule without degradation. The amount of total phenolic compounds in the supernatant solution was determined by the Folin–Ciocalteu method and the adsorption at 740 nm by a spectrophotometer. The percentage of encapsulation efficiency was calculated from the following equation:

$$\text{Encapsulation Efficiency (}\%)=\frac{w1‐w2}{w2}\times 100$$

In this equation, 1w is the amount of extract in the upper liquid, a certain amount of nanoencapsules, and 2w is the amount of extract added to prepare the same amount of nanoencapsules, which are expressed in milligrams of gallic acid per gram of plant.

### 2.7 Measuring particle size

Average diameter, particle size distribution, and specific surface area were measured using a laser light refraction device (Zetasizer Nano ZS, Malvern, UK). The average particle diameter was shown by the symbol 43d (volume‐length diameter) and was calculated according to the following equation. In this formula, zi will be the number of particles with diameter di (Joye et al., 2015).

$$D_{4,3}=\sum n_{i}d_{i}^{4}/\sum n_{i}d_{i}^{3}$$

### 2.8 Zeta potential measurement

A zetasizer (Zetasizer Nano ZS) was used to measure the zeta potential. The device comprises an electrochemical cell containing two electrodes. Samples are deionized with water in a ratio of 5:1 diluted and were placed in the tube. When voltage was applied, the negatively charged particles moved toward the positive electrode, and the particle velocity was measured (Joye et al., 2015).

### 2.9 Releasing the extract from the capsules in the laboratory

The release of the extract in vitro in a simulated stomach medium in the presence of pepsin enzyme will be performed according to the method of Chen et al., (2008). One gram of the nanocapsules of the whey–maltodextrin protein concentrate loaded with the extract was dissolved in a laboratory tube in 0.5 ml of 0.1 N hydrochloric acid and incubated at 37°C for 10 min at a rate of 100 rpm. Then, 0.2 ml of pepsin solution with a concentration of 1 mg/ml in hydrochloric acid 1/0 normal was added to start hydrolysis. Digestion was performed for 30 min, then the digested sample was centrifuged at 18,000 *g*, and a clear liquid supernatant was obtained. The amount of released extract was reported as a percentage of the total microencapsulated extract obtained from the microencapsulation efficiency.

### 2.10 Preparation of functional probiotic yogurt

First, standardized milk (containing 1.5% fat and 10.5% nonfat dry matter) was pasteurized at 90°C for 5 min. After homogenization, it was cooled to 43°C. At this temperature, yogurt starter culture (express 0/1 included *streptococcus thermophiles* and *Lactobacillus delbrueckii*, subspecies of *bulgaricus* were obtained from DVS CR401, Chr. Hansen Laboratory) (0.6 gr) and probiotic bacteria (*Lactobacillus casei*, subspecies of casei (PTCC 1608), and *Lactobacillus plantarum*, subspecies of plantarum (PTCC 1745) were prepared as pure strains from Organization of Scientific and Industrial Research of Iran (0.4 gr) and directly added to milk (800 gr; Keshavarzi et al., 2021). At the same stage, free and nano‐TPE were added at different levels (750 and 1,000 ppm) (control sample was considered without adding the extract). Then, incubation was performed at 43°C until pH = 4.7. After that, it was stirred for 3–5 min to get a completely uniform appearance. In the next step, the resulting product was packed in 100 g containers, and then the samples were stored in the refrigerator at 4°C. Finally, the tests were performed at intervals of 1, 8, and 15 days with three replications (Ghasempour et al., 2020).

### 2.11 PH determination

To measure pH, a pH meter was used based on the amount of free H+ ions in the sample. To measure the pH of the samples, before each operation, the pH meter was first calibrated by 24 and 7 buffers. PH changes were observed during incubation and the end of incubation and during the storage period in the refrigerator in 3 replications (Ghasempour et al., 2020).

### 2.12 Measurement of acidity

Acidity was measured by the titration method using a burette and 0.1 N sodium and phenolphthalein reagent. First, the yogurt sample was completely uniform. 10 grams of yogurt was removed from the container and poured into a 100‐ml glass beaker. 10 ml of distilled water was added to dilute it. Then, 3–4 drops of phenolphthalein solution were added to it and titrated with 0.1 N sodium. After the appearance of pale pink color, the volume of sodium was read from the Burette and entered in the following formula (AOAC, 2005).

$$\frac{V\times 0/9}{M}=\text{TA or acidity according to Dornick degree}$$

where in it:

V = volume of sodium hydroxide solution consumed in milliliters

M = sample weight

### 2.13 Syneresis

The specified weight of the yogurt cup was kept at a 45 ° angle in the refrigerator for 2 hr at 5°C to separate the yogurt water. Yogurt water separated from the surface was sucked using a syringe and weighed. Syneresis are calculated as one percent of the weight of yogurt juice to the initial weight of yogurt (Zainoldin & Baba, 2009).

$$y=\frac{a}{b}\times 100$$

*y* = percentage of synergy

*a* = separated yogurt water

*b* = initial weight of yogurt

### 2.14 Viscosity measurement

The apparent viscosity of the samples, based on the method of Ranadheera et al., (2012), was determined by a Brookfield viscometer. The sample temperature at the time of viscosity reading was 15°C and its volume was 100 ml. Spindle No. 2 and 0.5 rpm were used for 1 min. This test was performed for each sample in three replications. And viscosity was reported in centipoise (CP).

### 2.15 Investigation of yogurt antioxidant activity

Antioxidant activity was evaluated using the DPPH method, and the reduction of adsorption in DPPH solution and yogurt at 517 nm was evaluated using a spectrophotometer on days 1, 7, and 15 (Zainoldin & Baba, 2009).

### 2.16 Counting probiotic bacteria

1 ml of the homogenized sample was mixed with 9 ml of physiological saline and diluted to 10–10 and 10–10 concentrations, and then 1 ml of each dilution in 3 replications was transferred in a plate containing acidic MRS‐Bile agar culture medium, and after complete mixing, it was incubated in an anaerobic jar with incubation conditions for 72 hr at 37°C. After this time, the number of live *L. casei* and *L. plantarum* bacteria in the sample was determined (Institute of Standards & Industrial Research of Iran, 1992).

### 2.17 Sensory analysis of yogurt

Sensory analysis of yogurt was performed using a 7‐point hedonic scale (1‐very bad and 7‐excellent). Panelists were selected from among 10 graduate students in the food industry. Evaluated features include color, taste, texture, and general acceptance. Yogurt samples were coded with numbers and randomly tested for sensory tests. All samples were sensory analyzed by panelists at 7°C (after being removed from the refrigerator for 10 min), and mineral water was used to evaluate the taste of each yogurt sample (Won et al., 2017). It was performed in 3 replications and in all 3 replications on the first and 15th day of yogurt refrigeration time. After providing an explanation to the panelists, evaluation forms were provided to them and the panel test was done.

### 2.18 Statistical analysis

All experiments were performed in a completely random experimental design with three replications, and the results were reported as mean (average) with standard deviation. Statistical analysis of treatments was performed by analysis of variance table (ANOVA) using IBM SPSS Statistics 22.0 (IBM SPSS, Inc.). Duncan test at the level of 0.05 was used to express the significant difference between the averages, and figures were drawn with Microsoft Excel software.

## 3 RESULTS AND DISCUSSION

### 3.1 Phenolic and flavonoid compounds

Phenolic compounds in fruits and vegetables have attracted the attention of many researchers due to its high potential for antioxidant activity. Phenolic compounds inhibit free radical activity by donating hydrogen atoms (Tometri et al., 2020). These compounds include substances such as flavonoids, flavonol, anthocyanins, anthraquinones, and their derivatives. Among phenolic compounds, flavonoids are the most potent antioxidant compounds. Flavonoids are potent inhibitors of hydroxyl and peroxide radicals. Increasing the number of hydroxyl groups is directly related to the antioxidant properties of phenolic compounds (Rashidaie Abandansarie et al., 2019). The total amount of phenolic compounds in different treatments of TPE which was extracted by ultrasound was equal to 890.04 ± 2.18 mg gallic acid per gram of extract, and the amount of flavonoid compounds was equal to 512.76 ± 2.08 mg per gram extract. Farzaei et al., (2014) reported the amounts of phenolic compounds in different parts of the TPE using the Soxhlet method as 560.7–292.3 mg gallic acid per gram of extract. The reason for the higher amounts of phenolic compounds in this study may be due to differences in the extraction method in these studies. In their study, the solvent method was used, but in this study, the ultrasound method was used. In fact, ultrasound waves facilitate both stages of the extraction process, that is, texture swelling, as well as the removal of compounds from it by creating pores in the cell wall and improving the diffusion and mass transfer, in which this increase in the solvent permeability in cell tissues is caused by the mechanical effects of ultrasound, and thus living cells are destroyed by these waves and release their internal materials better and easier (Bahrami Feridoni & Khademi Shurmasti, 2020; Kadam et al., 2015).

### 3.2 Assessment of DPPH free radical activity

**FIGURE 1:**
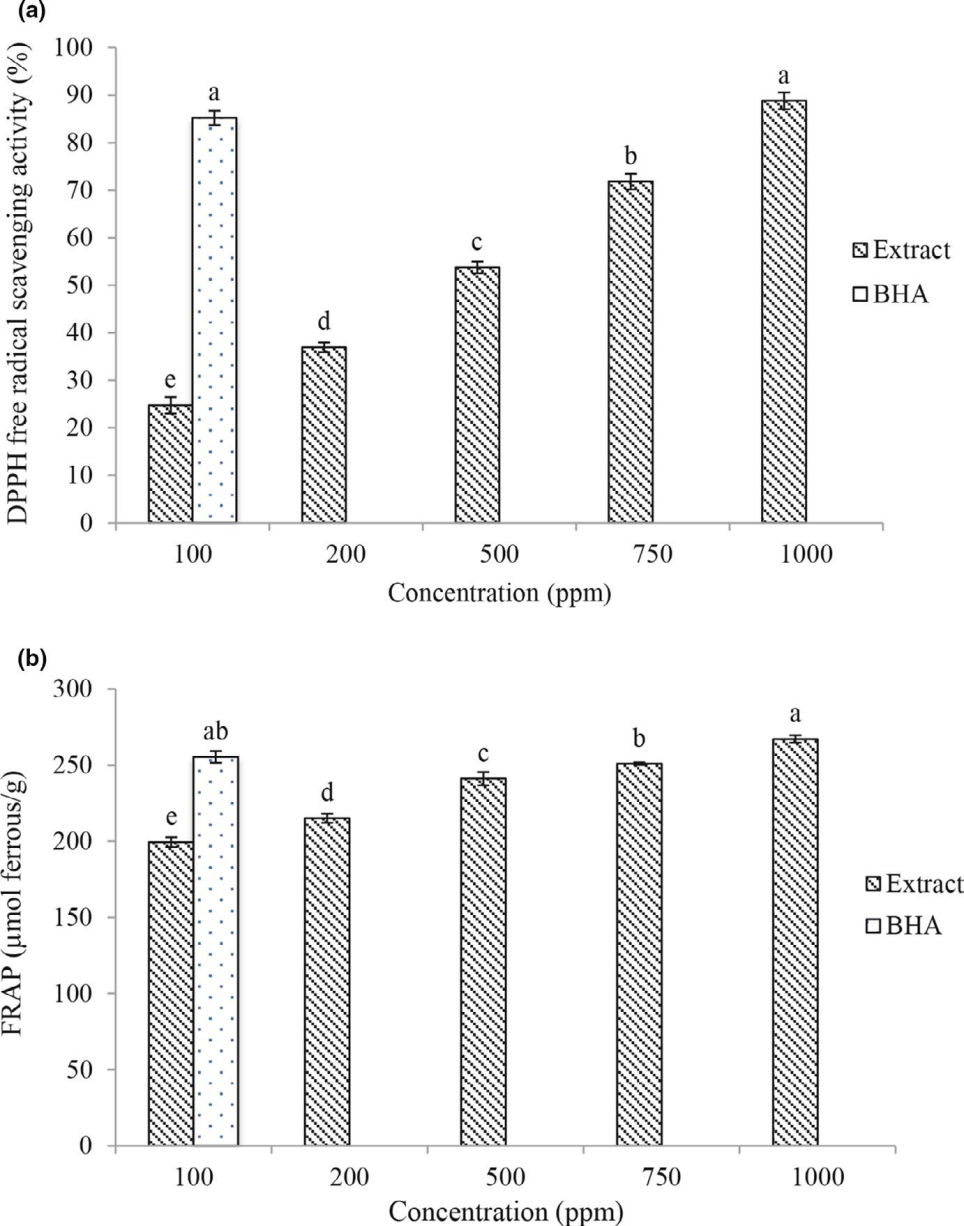
The antioxidant activity [DPPH (a) and FRAP (b)] of *Tragopogon Collinus* extract

Assessing the DPPH free radical activity is a simple way to determine the antioxidant activity of extracts. This compound is a free radical with the central nitrogen atom that stabilizes by reducing and producing a molecule that changes color from purple to yellow. This process is analyzed using spectrophotometry (Ahmad et al., 2020). The results of this study showed that the amount of DPPH free radical activity (Figure 1a) increased with increasing concentration (*p* < .05). The extract had the highest antioxidant activity at a concentration of 1,000 ppm, and the amount of DPPH free radical activity at this concentration was not significantly different from the synthetic antioxidant BHA (*p* < .05). Herbal extracts have antioxidant activity due to their phenolic compounds that have antioxidant activity and high capacity for donating hydrogen or electron atoms and free electrons. With an increase in the concentration of phenolic compounds or the degree of hydroxylation of phenolic compounds, the radical inhibitory activity of the extract has increased (Rashidaie Abandansarie et al., 2019; Tometri et al., 2020). Farzaei et al., (2014) also stated that TPE has antioxidant properties and can inhibit DPPH free radicals. They also stated that phenolic compounds play an important role in inhibiting DPPH free radicals.

### 3.3 Evaluation of ferric reducing power (FRAP)

Ferric reducing power measurement is a simple and easy method and measures the regenerative power of Fe in herbal extracts. The presence of reducing agents in the extract reduces ferric ions (Fe^+3^) to ferrous ions (Fe^+2^). This reduction is measured by blue‐green color at a wavelength of 700 nm (Sopee et al., 2019). The results of this study showed that with increasing concentration, the amount of FRAP (Figure 1b) increases. The extract had the highest antioxidant activity at a concentration of 1,000 ppm, and the amount of FRAP at this concentration was significantly higher than the synthetic antioxidant BHA (267.16 μmol Fr/g) (.05 > *p*). The antioxidant activity of the reducing agents in the extract is based on breaking the chain reactions of free radical formation by donating electrons or hydrogen atoms. Also, during the extraction process, other compounds which can be highly soluble in water and alcoholic solutions enter the extracts along with phenolic compounds, and since some of these compounds, such as ascorbic acid, proteins, and sugars themselves are electron donors, a higher percentage of trivalent ferric ions are reduced by electron adsorption and therefore the adsorption intensity of the solution increases. As a result, increasing the concentration of the extract increases all of these compounds and consequently the ferric reducing power also increases (Bahrami Feridoni & Khademi Shurmasti, 2020; Sannigrahi et al., 2010).

### 3.4 Investigation of antimicrobial activity

One of the criteria used by most researchers to measure the antibacterial activity of antimicrobial agents is to measure the minimum inhibitory concentration and the minimum lethal concentration. According to the results (Table 1), TPE had antimicrobial properties against both bacteria. Regarding the action of herbal extracts in the death of pathogenic bacteria, it has been commented that one of the important properties of these substances and their compounds is their hydrophobicity, which causes them to distribute in the lipid parts of the cell wall and mitochondria of the bacterium, causing changes and destruction of the structure and their greater permeability, followed by many ions and other vital elements of the cell leak out, which eventually leads to the death of the bacterium (Brusa et al., 2013). According to MIC and MBC results, *Staphylococcus aureus* was more sensitive to the extract than *Escherichia coli*. Numerous reports have stated that gram‐positive bacteria are more sensitive to antibacterial compounds than gram‐negative bacteria, and this high sensitivity of gram‐positive bacteria is due to the absence of a lipopolysaccharide cell wall, while in gram‐negative bacteria it may prevent active compounds from entering the cytoplasmic membrane. (Rashidaie Abandansarie et al., 2019).

**TABLE 1:** Antibacterial activity [MIC (a) and MBC (b)] of *Tragopogon Collinus* extract

Treatment	MIC (ppm)	MBC (ppm)
*Staphylococcus aureus*	183.33 ± 14.43^b^	316.33 ± 25.66^b^
*Escherichia coli*	283.66 ± 25.66^a^	416.66 ± 14.43^a^

Note

^a,b,c^Different small letters in the same row represent significant difference (*p* < .05).

### 3.5 Properties of nanoencapsules

Particle size and particle size distribution are of particular importance in determining the properties of colloidal systems. The values and stability of these two parameters play an important role in determining the stability of the colloidal carrier system and its microencapsulation efficiency. Results related to the particle size of the encapsulated extract by maltodextrin–WPC in this study were 93.87 ± 1.79 nm. According to the results, the microencapsulated extract is small. Nano‐extracts with smaller size are more stable due to their higher resistance to gravity due to Brownian motion (Fathi et al., 2012).

Also, the results related to the microencapsulation efficiency of extract microencapsulation by maltodextrin–WPC in this study were equal to 64.35 ± 1.87%. Bahrami Feridoni and Khademi Shurmasti (2020) stated that the particle size of microencapsulated extract of sour tea with maltodextrin–WPC is 139.03 ± 2.76 nm; the microencapsulation efficiency was 67.30 ± 1.37.

The presence of charged surfactants, such as ionic surfactants, polysaccharides, and proteins in emulsion results in different electrical charges at the droplet surface. The zeta potential indicates the magnitude of the electric charge and the electrostatic reactions between the particles in the suspensions. The higher the zeta potential the more the repulsive forces between the droplets and the fewer tendencies to stick together, in which the emulsion droplets repel each other, resulting in a stable system. The zeta potential affects the aggregation and bonding of nanoparticles and plays an important role in their stability. (Rydström Lundin, 2012). The results related to zeta potential were equal to 18.99 ± 0.21 mV microencapsulation extract by maltodextrin– WPC. Colloidal depressions of systems with zeta potential from +30 to −30 mV are considered stable (Sebaaly et al., 2016). The high level of zeta potential in the nano‐extract indicates the high stability of the generated nanoparticles. According to previous researches, zeta potential increases with decreasing liposome size (Rasti et al., 2012; Sebaaly et al., 2016).

The results related to the releasing rate (Table 2) of the extract from the nanocapsules during the storage period showed that over time, the releasing rate of the extract increased significantly (*p* < .05). During storage, the decomposition of the nanocapsules occurs gradually time, which leads to the release of capsule materials. This may be due to a decrease in the stability and cohesion of the biopolymers used as the coating material and an increase in the diffusion rate under environmental stresses of temperature and humidity. The absorption of moisture from the environment in which the nanocapsules are placed leads to swelling of the biopolymer walls and at the same time reduces the glass transition temperature. As a result, the coherence and entanglement of biopolymer chains is reduced, and the rate of molecular motion of the microencapsulation particles is increased. These results are consistent with the results of Mohammadi et al., (2016). While examining the characteristics of nanocapsules prepared from whey protein and pectin in the microencapsulation of olive leaf polyphenols, they stated that with the storage time up to day 20, the release of phenolic compounds from the capsule increases.

**TABLE 2:** Releasing rate of the extract from the nanocapsules during the storage

Storage time (time)	0	8	16	24	32	40
Releasing rate	4.59 ± 0.15^f^	22.98 ± 0.58^e^	43.34 ± 0.39^d^	59.95 ± 3.27^c^	69.98 ± 2.55^b^	78.35 ± 1.98^a^

### 3.6 Measuring pH and acidity of yogurt

**FIGURE 2:**
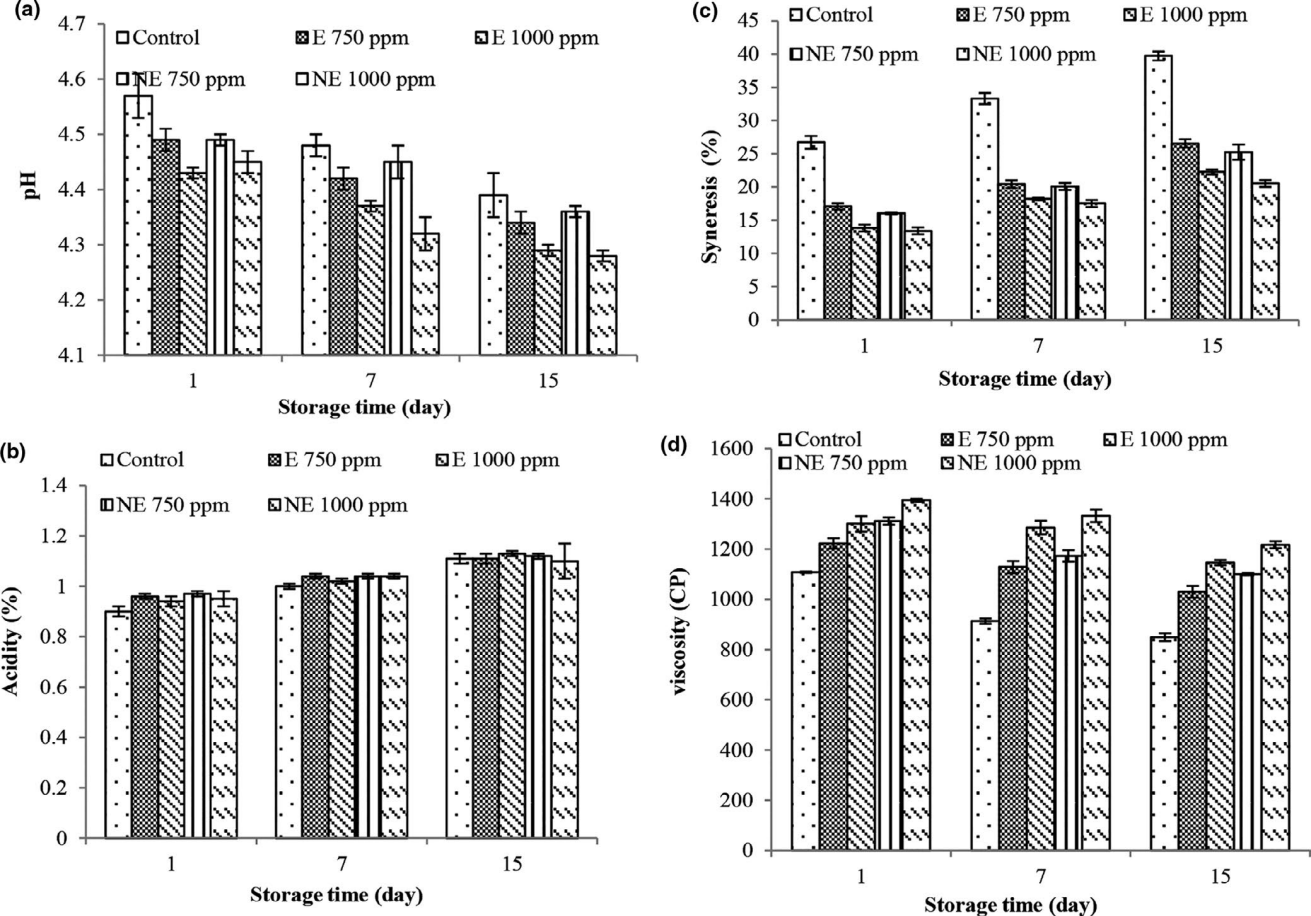
Changes in pH (a), acidity (b), syneresis (c) and viscosity (d) of different treatment during storage

PH and acidity values are important factors in preparing a probiotic product; decreasing the pH during the storage period of the product is associated with increased production of acid by bacteria and the most acid produced is lactic acid. If the amount of this acid is too much, it affects the taste of the product and creates unfavorable conditions for the product (Bruno et al., 2002). The results related to the pH values (Figure 2a) and acidity (Figure 2b) during storage showed that pH values in all treatments decreased with increasing time, and the acidity values increased (*p* < .05); the cause of this phenomenon is more related to the production of lactic acid by bacteria of lactic acid, which can produce four molecules of lactic acid from two molecules of lactose (Esfandiari & Moslehishad, 2019). The trend of decreasing pH and increasing acidity during storage is to be expected, which has been mentioned in most related studies (Esfandiari & Moslehishad, 2019; Won‐Young et al., 2020).

The results related to the pH and acidity values of yogurt showed that with the addition of TPE, the pH values decreased and the acidity values increased, but the pH and acidity values in the treatments containing the extract and nano‐extract did not differ at different times. The presence of the extract seems to increase the metabolic activity of yogurt bacteria (Amirdivani & Salihih Hj Baba, 2012). In the early hours of incubation, by increasing the substrate available for the growth of microorganisms, the metabolic activity of bacteria increases and it causes the pH value decrease and the acidity value in samples containing the extract increase. Esfandiari and Moslehishad, (2019) reported that adding rice bran and lettuce extract to yogurt decreased pH and increased yogurt acidity. Tang et al., (2020) also reported that adding cinnamon bark and wood extract in free form and nanoencapsulated in fermented milk decreased pH and increased yogurt acidity. The treatments containing the extract and nano‐extract did not differ from each other.

### 3.7 Investigation of yogurt syneresis and viscosity

One of the major disadvantages of yogurt is syneresis, which leads to the appearance of serum or whey on the surface of yogurt and shrinkage of the three‐dimensional structure of the pH protein network. Syneresis in yogurt occurs due to changes that decrease the binding power of whey proteins and this process causes the water in yogurt to drain out. Results related to syneresis (Figure 2c) changes over time increased in all treatments (*p* < .05). The phenomenon that occurred during syneresis is not fully understood, but it is agreed that increased syneresis with storage time is associated with severe rearrangements of the casein network that increases serum output. Rearrangements increase particle connections, thus the network tends to compress and shrink, separating serum (Ramirz‐Santiago et al., 2010). The highest values of syneresis were observed in the control treatment and the lowest values of syneresis were observed in treatments containing extract and nano‐extract with a concentration of 1,000 ppm that these two treatments were not different during the storage period (*p* < .05). Hydrocolloids that have high water absorption and this water absorption, increased with increasing concentration of the extract (Temiz et al., 2014). The release of whey from yogurt is affected by the physical properties of yogurt during the storage period. The addition of the extract increases the dry substance and as a result the tissue hardens and decreases syneresis (Coda et al., 2012). The lowest values of viscosity were observed in the treatment of nano‐extract with a concentration of 1,000 ppm. The results are in agreements with the results of Afzaal, Khan, et al., (2019).

Yogurt viscosity is an important property that affects its quality. Stirred yogurt is a homogeneous and viscous substance, which is affected by various factors such as the incubation temperature, fat and casein content, heat treatment of milk, milk acidity, type of starter culture and additives. The results related to viscosity (Figure 2d) changes in all treatments decreased over time. The decrease in viscosity during the storage period may be due to changes in the protein–protein binding in the three‐dimensional protein network of our samples. The addition of extract and nano‐extract to yogurt increased the viscosity and on the 15th day of storage, the highest values of viscosity were observed in the treatment of nano‐extract with a concentration of 1,000 ppm and the lowest values were observed in the control treatment. The results are in agreements with the results of Afzaal, Saeed, et al., (2019). It can be said that most hydrocolloids increase their viscosity due to their water absorption properties (Temiz et al., 2014). Won‐Young et al., (2020) also reported that by adding olive leaf extract to yogurt, it reduced syneresis and increased the viscosity of yogurt; additionally, with increasing storage time, the amount of syneresis increased and the viscosity decreased.

### 3.8 Investigation of antioxidant activity in yogurt

**FIGURE 3:**
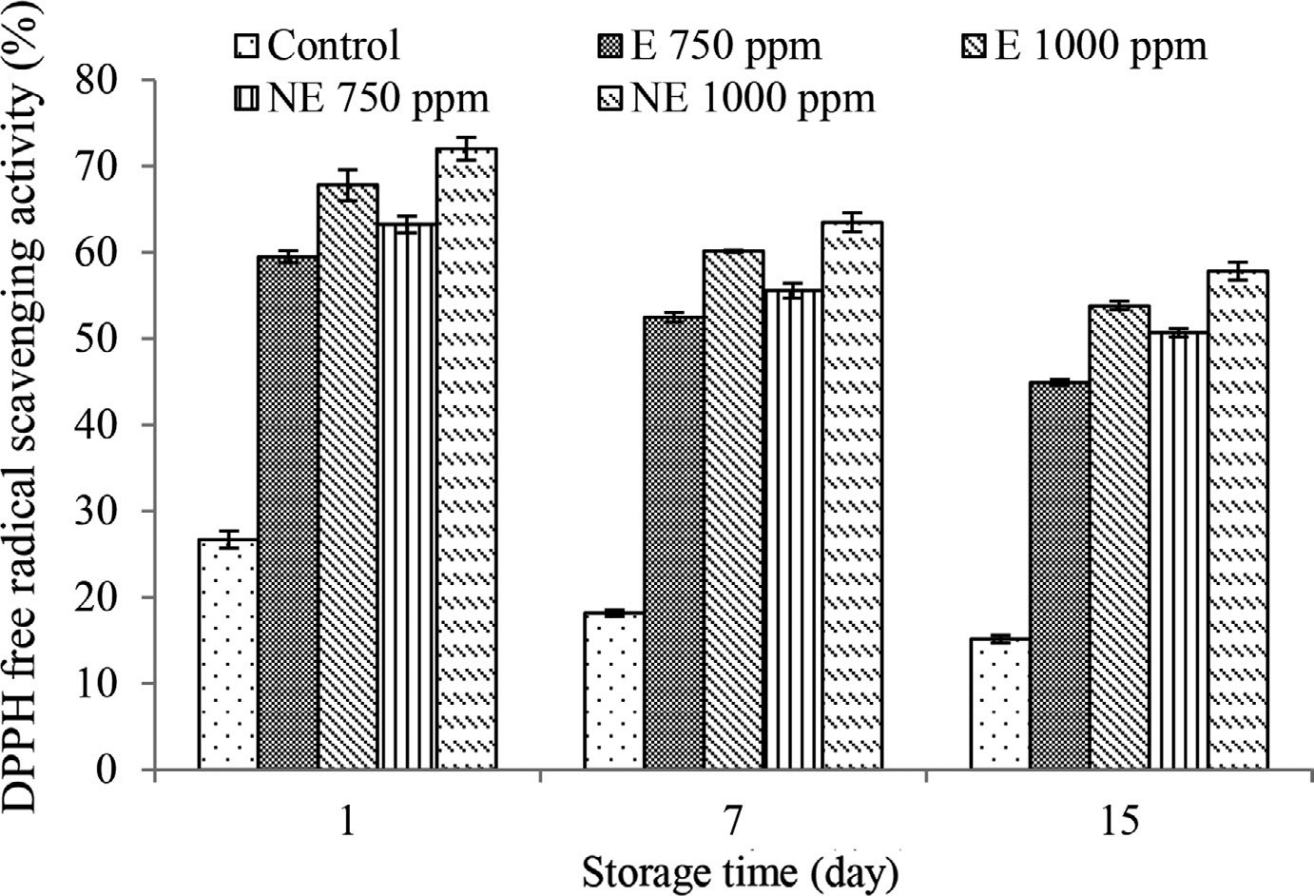
Changes in antioxidant activity of different treatment during storage

The results of this study showed that the levels of antioxidant activity (Figure 3) decreased in all treatments (*p* < .05). According to researches, this decrease may be due to the effect of beta‐glucosidase, lactase and peroxidase enzymes during storage on the breakdown of these compounds. (Amaretti et al., 2013). Decreased antioxidant activity of yogurt during storage has also been reported by other researchers (Esfandiari & Moslehishad, 2019; Novzari et al., 2019; Won‐Young et al., 2020).

In all storage days, the highest levels of antioxidant activity were observed in the treatment of nano‐extract with a concentration of 1,000 ppm and the lowest values were observed in the control treatment (*p* < .05). The reason for the higher antioxidant properties in treatments containing TPE is due to the presence of phenolic compounds in the extract. Many studies show that high phenolic compounds are the main reason for the high antioxidant activity; because based on the available evidence, there is a direct relationship between the amount of phenolic compounds and antioxidant power in plants (Maleki et al., 2016). Microencapsulation also increased the amount of DPPH free radical activity in all treatments. Microencapsulation protects the used hydrocolloids from environmental factors such as pH, oxygen, light, etc. Also, volatile molecules remain stable with this method and protect them from oxidative, optical and volatile changes. Therefore, microencapsulation has more potential to increase bioavailability, improve emission control, and accurately target biological compounds because of improving antioxidant activity (Ezhilarasi et al., 2013). Tang et al. (2020) reported that cinnamon bark and wood extract in free form and nanocapsules in fermented milk increased the free radical scavenging of DPPH in yogurt. Treatments containing nano‐extract had higher antioxidant activity.

### 3.9 Investigation of probiotic bacteria viability

**FIGURE 4:**
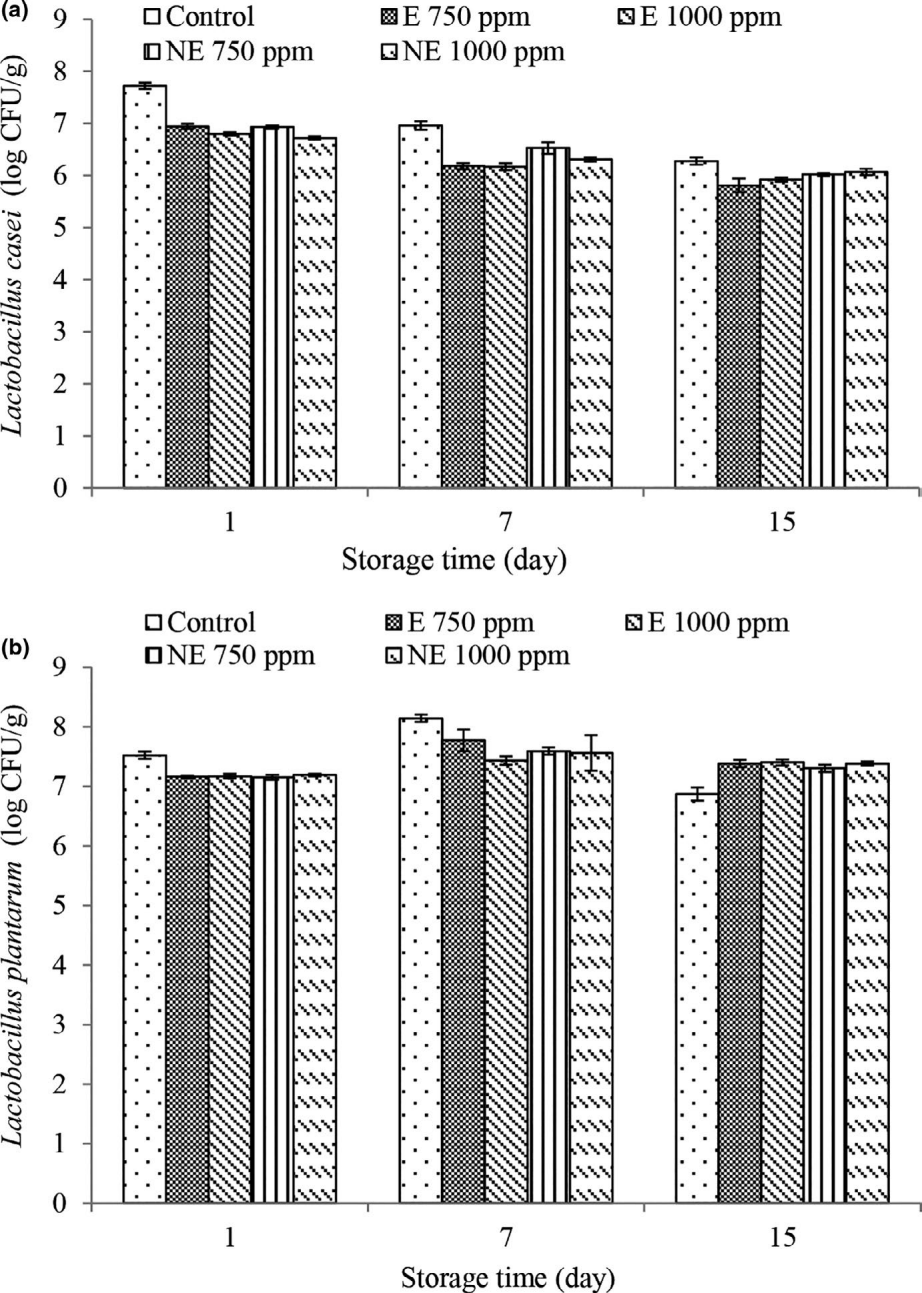
Changes in *Lactobacillus casei* (a) and *Lactobacillus plantarum* (b) of different treatment during storage

As probiotic bacteria affect human health, these bacteria must be present in the required number until the time of consumption in the product, thus the number of live bacteria during the storage period was examined. According to the opinion of most scientists, at least 10^6^ cells per gram of product are needed to create the health effects of probiotics. Viability rates of *L. casei* (Figure 4a) decreased in all treatments with increasing storage time, this reduction increased in the control treatment (*p* < .05). In fact, the addition of the extract slowed down the bacterial reduction process during the storage period. All treatments had rates above 10^6^ cells per gram until the end of the storage period. Viability rates of *L. plantarum* (Figure 4b) increased in all treatments on the 7th day and then decreased significantly on the 15th day. Also, the highest viability rates of *L. plantarum* were observed at the beginning of the storage period in the control treatment and at the end of the storage period in treatments containing the extract and nano‐extract (*p* <.05). Also, at the end of the storage period; the lowest rates were observed in the control treatment. Extensive research has shown that the number of probiotic bacteria is significantly reduced during refrigerated storage, especially in acidic environments. In fermented products such as yogurt, low pH and high acidity of the product and on the other hand, cultures of nonprobiotic lactic bacteria with probiotic starters cause faster death of probiotics during storage, especially in the final stages (Sendra et al., 2010). However, as mentioned above, the addition of the extract had a positive effect on the viability of probiotic bacteria. This is due to the phenolic compounds in herbal extracts that have a stimulating role and improve the growth of yogurt starter bacteria (Oh et al., 2016) and probiotic bacteria (Marhamatizadeh et al., 2013). Novzari et al., (2019) reported that the addition of savory extract to yogurt slowed down the reduction process of *Lactobacillus acidophilus* during storage in yogurt.

### 3.10 Sensory evaluation of the first day

**FIGURE 5:**
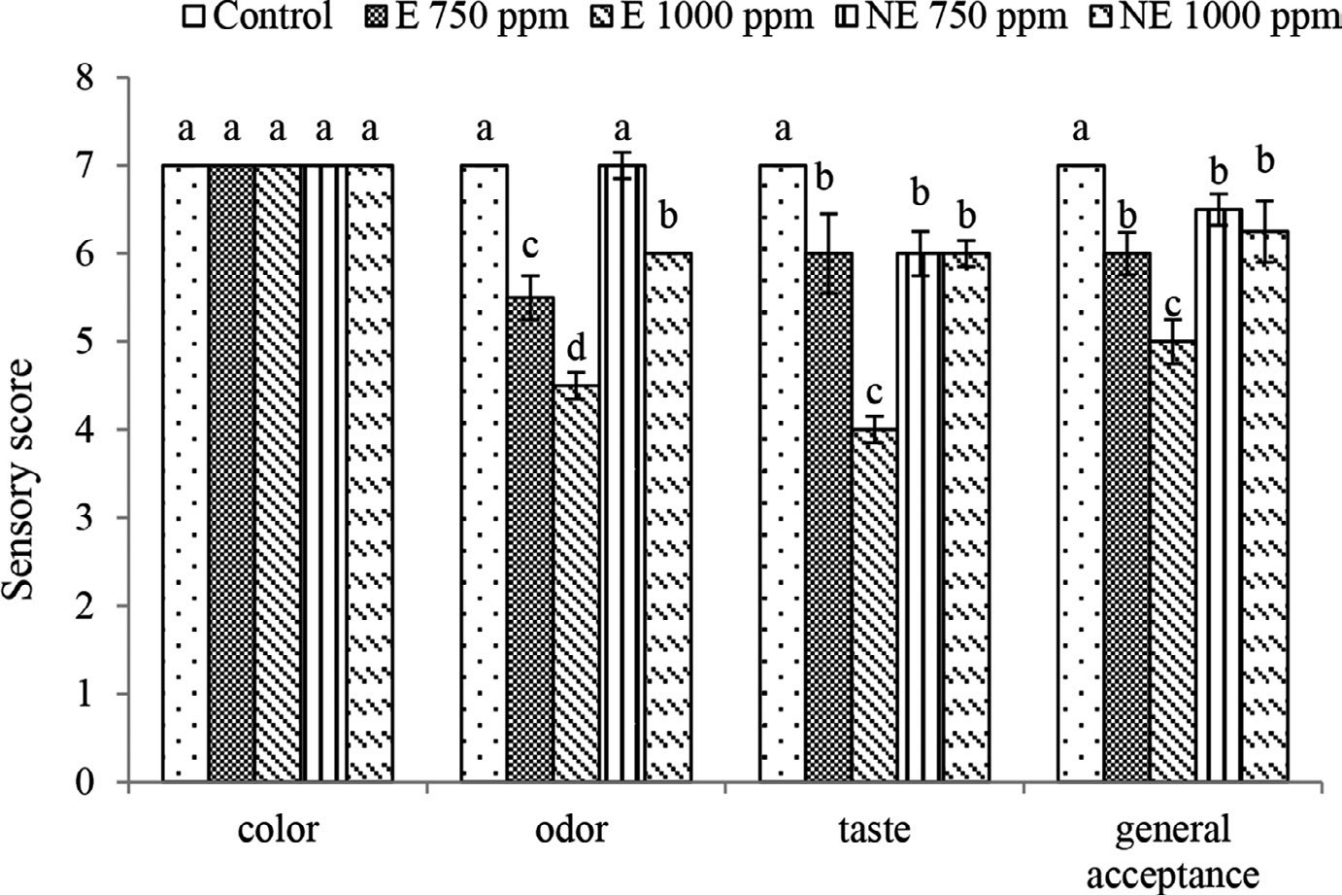
Sensory evaluation in different treatments at the beginning of storage

Sensory properties are the main factors in accepting or rejecting many products and gaining satisfaction from their consumption. According to the results of statistical analysis (Figure 5), adding preservatives did not change the color of yogurt. In relation to taste, odor and general acceptance, with the addition of extracts, the sensory score was significantly reduced and with increasing concentration of the extract, a lower sensory score was observed. Generally, the sensory score in the treatments containing nano‐extract was higher than the treatments containing the extract. Won‐Young et al., (2020) reported that the addition of olive leaf extract to yogurt, reduced the sensory score of yogurt and the addition of olive leaf extract to a concentration of 2% was approved by evaluators, but a concentration of 4% was not approved by sensory evaluators. Esfandiari and Moslehishad, (2019) also stated that adding rice bran and lettuce extract to yogurt reduced the sensory score of yogurt.

## 4 CONCLUSION

The results of this study showed that the addition of TPE to yogurt affects the physicochemical properties. In most cases, better results were observed in treatments containing nano‐extract. Yogurt containing the extract was acceptable in terms of sensory properties and the treatments containing nano‐extract had a higher sensory score compared to the control treatment. Therefore, it seems that the production of yogurt containing nano‐TPE, as a functional food, provided a new choice for consumers of dairy products. These products not only have the desired taste, but also their consumption provides good nutritional properties.

## CONFLICT OF INTEREST

The authors declare that they do not have any conflict of interest.

## ETHICAL APPROVAL

Human and animal testing is unnecessary in this study.

## INFORMED CONSENT

Written informed consent was obtained from all participants.

## Data Availability

Data that support the findings of this study will be available from corresponding author upon reasonable request.
